# Cosmetic Appearance After Eye Loss: The Role of Prosthesis Motility and Eyelid Function

**DOI:** 10.3390/jcm15208054

**Published:** 2026-10-16

**Authors:** Yoav Vardizer, Oren Tomkins-Netzer, Yinon Shapira

**Affiliations:** 1 The Ruth and Bruch Rappaport Faculty of Medicine, Technion—Israel Institute of Technology, Haifa 3525433, Israel; 2 Department of Ophthalmology, Bnai Zion Medical Center, Haifa 31048, Israel; 3 Department of Ophthalmology, Carmel Medical Center, Haifa 3436212, Israel

**Keywords:** anophthalmic socket, cosmetic outcomes, appearance, ocular prosthesis, motility, artificial eye, eyelid symmetry

## Abstract

**Background/Objectives:** We aimed to clarify which clinical and prosthetic features are most strongly associated with cosmetic appearance after eye loss. **Methods:** This prospective cross-sectional study included 107 patients with unilateral anophthalmia wearing acrylic prostheses. Examiner-rated cosmetic outcomes—eyelid symmetry, orbital fullness, prosthesis recognizability, and overall appearance—were graded by three independent raters. Candidate predictors associated in univariate analysis (*p* ≤ 0.10) were entered into blockwise multivariable regression across four domains: clinical background, socket and eyelid anatomy, prosthesis alignment and motility, and prosthesis dimensions. **Results:** Levator function (β = 0.19, *p* = 0.02) and prosthesis motility (β = 0.68, *p* < 0.001) predicted eyelid symmetry (R^2^ = 0.61). Orbital fullness was predicted by relative exophthalmometry (β = 0.27, *p* = 0.039) and motility (β = 0.34, *p* = 0.002; R^2^ = 0.34). Recognizability was predicted by motility (β = 0.55, *p* < 0.001) and history of additional periocular surgery (β = −0.22, *p* = 0.008; R^2^ = 0.46). General appearance was predicted by lagophthalmos (β = −0.38, *p* < 0.001) and motility (β = 0.35, *p* < 0.001; R^2^ = 0.69). Prosthesis dimensions were not independently associated with any of the outcomes. **Conclusions:** Cosmetic appearance after eye loss was most strongly associated with dynamic anatomical features. Prosthesis motility consistently predicted all appearance domains, while upper-eyelid function was associated with symmetry and overall impression. In contrast, background and static anatomical or prosthesis features showed limited independent associations once dynamics were accounted for. These findings highlight prosthesis motility and eyelid dynamics as important considerations in rehabilitation and routine follow-up, while cosmetic outcome assessment should incorporate dynamic features alongside conventional static measures.

## 1. Introduction

After eye loss, cosmetic rehabilitation becomes central to patient recovery and social reintegration. Although specialized services manage complex cases, many prosthesis wearers are followed primarily by general ophthalmologists—often for monitoring the fellow eye—who still encounter appearance-related concerns during routine care.

Cosmetic outcomes after eye removal and prosthesis fitting have traditionally been evaluated in the context of well-recognized complications such as orbital volume loss, enophthalmos, superior sulcus deformity, eyelid malposition, and socket contraction [1,2,3,4,5]. Yet many anophthalmic patients present with only subtle anatomical changes, and most prosthetic wearers do not require complex surgical rehabilitation [6]. Despite this, agreement remains limited on what constitutes cosmetic success in routine prosthetic care [7].

Most previous studies explored patient experiences and self-perceived outcomes [8,9,10,11,12,13,14], whereas few have examined how others judge appearance [7,15,16]. Consequently, observer-based evaluations are uncommon, and the clinical and prosthetic parameters that determine cosmetic appearance remain unclear.

The cosmetic result of an anophthalmic socket reflects a complex interplay between orbital anatomy, eyelid mechanics, and prosthesis fit and movement. Jordan and Klapper [17] described a widely accepted set of features characterizing the “ideal” anophthalmic socket: a centrally placed implant of adequate volume that transmits motility, healthy conjunctiva with sufficiently deep fornices, symmetric eyelid and sulcus contours, and a prosthesis resembling the fellow eye in position and appearance. While these criteria are grounded in clinical experience and consensus, their relative influence on cosmetic impressions remains unquantified.

This study systematically evaluated clinical, anatomical, and prosthetic variables—including background characteristics, eyelid and socket anatomy, dynamic features such as motility and eyelid function, and prosthesis dimensions—to identify independent predictors of examiner-rated cosmetic outcomes across four domains: eyelid symmetry, orbital fullness, prosthesis recognizability, and overall appearance. The aim was to identify which clinical and prosthetic factors are most strongly associated with cosmetic appearance after eye loss.

## 2. Subjects and Methods

### 2.1. Study Design and Participants

This prospective, cross-sectional study was conducted at a tertiary ocular prosthetics clinic. Between 2010 and 2011, we recruited consecutive adult anophthalmic patients attending routine follow-up visits who met the eligibility criteria. The original study eligibility criteria were age ≥ 18 years, use of a unilateral custom-made acrylic (polymethyl methacrylate [PMMA]) ocular prosthesis, and the ability to complete a self-administered questionnaire. Questionnaire-based outcomes were not analyzed in the present study. Patients with bilateral or congenital anophthalmia, or those wearing scleral shells, were excluded from the study.

The sample size was determined by the number of eligible participants recruited during the study period; no a priori sample-size calculation was performed.

Institutional review board approval was obtained for this study (Approval No. 0023-09-BNZ), and all participants provided written informed consent before enrollment. This study adhered to the tenets of the Declaration of Helsinki.

### 2.2. Data Collection

Demographic and clinical background data were collected for each participant. Each then underwent a structured clinical assessment by three independent examiners (an oculoplastic surgeon, an ocularist, and a senior ophthalmologist). Objective clinical measurements were performed by the oculoplastic surgeon. Clinical variables requiring subjective or semi-quantitative assessment were rated independently by all three examiners, without knowledge of the other examiners’ ratings, and the mean of the three ratings was used for analysis after assessment of inter-rater agreement.

The evaluation included eyelid and socket parameters: marginal reflex distance-1 (MRD1), levator function, lagophthalmos, lower eyelid laxity, relative exophthalmometry, socket support contour, inferior and superior fornix depth, inferior fornix position relative to the inferior orbital rim, and socket contracture. Lower-eyelid laxity was assessed using the snap-back test in comparison with the fellow side and recorded as present or absent. Relative exophthalmometry was measured using a Hertel exophthalmometer and defined as the difference in anterior projection between the prosthetic and fellow sides (prosthetic minus fellow eye). Socket support contour was clinically categorized as relatively rounded or flat. Superior and inferior fornix depths were measured by gently pulling the corresponding eyelid forward and measuring with a ruler from the eyelid margin to the transition of the conjunctival cul-de-sac to the bulbar conjunctiva. Socket contracture was graded on a three-point clinical scale: 3 = normal conjunctival surface, 2 = mild socket contraction with some restriction of eyelid closure, and 1 = severe socket contraction with marked restriction of eyelid closure.

Prosthesis-related appearance parameters were recorded, including prosthesis motility and primary gaze alignment. Prosthesis motility was graded relative to the fellow eye on a five-point horizontal excursion scale: 1 = minimal or no movement, 2 = <50% of fellow-eye excursion, 3 = approximately 50%, 4 = >50%, and 5 = almost full or near-normal excursion. Primary-gaze alignment was assessed using the Hirschberg corneal light reflex, comparing the position of the light reflex relative to the prosthetic pupil with that of the fellow eye.

Structural characteristics of the ocular prosthesis were evaluated after removal. Volume was measured using water displacement. Central thickness (T) was measured with a caliper at the center of the prosthesis, and posterior apex height (D) was measured from the plane of the prosthesis edges to the point of maximal posterior projection. The posterior projection index was calculated as the ratio D/T.

### 2.3. Outcomes

The three examiners also rated five appearance-related domains: prosthesis motility, eyelid position symmetry, orbital fullness, prosthesis recognizability, and general cosmetic appearance.

Eyelid position symmetry was graded on a five-point scale (1 = completely asymmetric; 2 = >3 mm difference; 3 = 1–2 mm difference; 4 = ≤1 mm difference; 5 = completely symmetric). Orbital fullness was graded from 1 (very deep superior sulcus) to 5 (normal fullness), with intermediate grades reflecting increasing degrees of volume deficiency. Prosthesis recognizability was graded on a four-point scale according to the distance or degree of inspection at which the prosthesis was considered recognizable: 1 = recognizable from a distance; 2 = recognizable at conversational distance; 3 = recognizable only on close inspection of the affected side; and 4 = unlikely to be recognized. General cosmetic appearance was graded from 1 (poor) to 10 (good). Thus, higher scores consistently represented a better cosmetic result.

### 2.4. Statistical Analysis

Inter-rater agreement was assessed using Kendall’s coefficient of concordance. Univariate analyses were first performed to identify variables associated with each cosmetic outcome. Associations with continuous predictors were tested using Spearman’s rank correlation; comparisons for categorical predictors used the Mann–Whitney U test or the Kruskal–Wallis test, as appropriate. Variables with *p* ≤ 0.10 were retained as candidates for multivariable modeling.

Although individual examiner ratings were ordinal, the outcomes entered into the regression models were mean scores across three independent examiners, yielding quasi-continuous measures that were analyzed using multiple linear regression.

To address the large number of potential predictors relative to the sample size, we used blockwise multiple regression. Blockwise modeling was chosen a priori to accommodate a relatively large set of conceptually distinct predictors within a moderate sample size and to structure variable selection within clinically relevant domains [18]. Variables associated with each cosmetic outcome in univariate analysis (*p* ≤ 0.10) were entered into domain-specific multiple linear regression for the four predefined blocks: (A) clinical background, (B) socket and eyelid findings, (C) prosthesis alignment and motility, and (D) prosthesis structural dimensions. Within each block, variables with *p* < 0.05 were retained. These retained variables were then entered simultaneously into a final integrated multivariable linear model for each outcome to estimate their independent contributions. Standardized regression coefficients (β) were used to express the relative independent contribution of predictors within each model. To allow direct comparison of explanatory performance across predictor blocks, the block models were additionally repeated within a common complete-case subset for each cosmetic outcome. Adjusted R^2^ was used for these comparisons to account for differences in the number of predictors across blocks.

Model assumptions were assessed using standard residual diagnostics, including Q–Q plots and residual-versus-fitted plots. Multicollinearity in the final integrated models was assessed using variance inflation factors (VIFs).

Analyses were performed using available data, with complete-case analysis for each regression model; no imputation of missing values was performed. Variable-level missingness is reported in Table S1, and the sample size of each block and final integrated model is reported in Table 1, Table 2, Table 3 and Table 4. Patients included and excluded from each final complete-case model were compared on available baseline characteristics to assess potential selection related to missing data.

All analyses were conducted using StatSoft Statistica software, version 12 (StatSoft, OKC, Tulsa, OK, USA).

**Figure 1:**
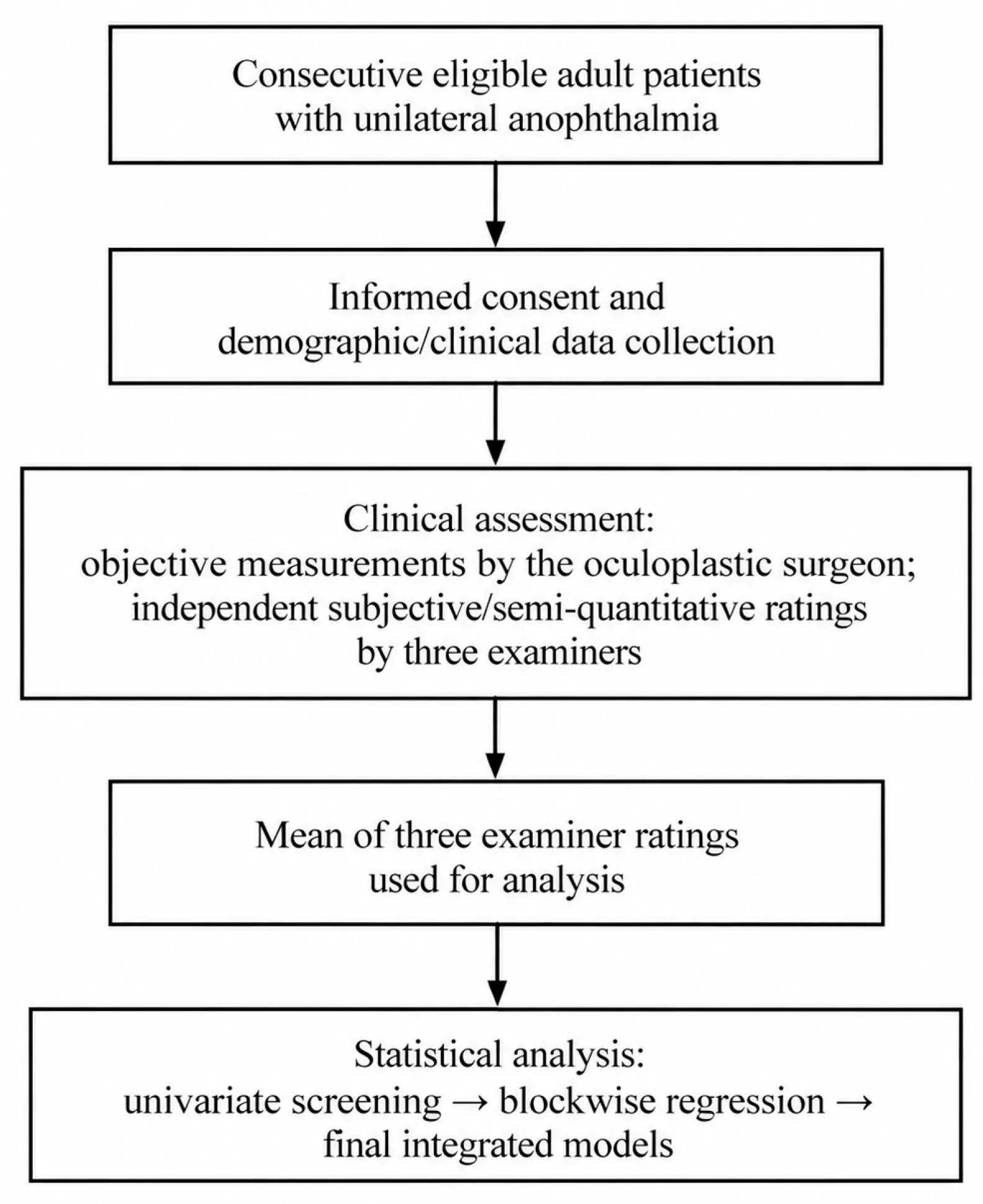
Study workflow. Overview of participant recruitment, clinical assessment, examiner ratings, and statistical analysis.

The overall study workflow is summarized in Figure 1.

## 3. Results

For all clinical and appearance-related variables requiring subjective or semi-quantitative examiner assessment, Kendall’s W ranged from 0.42 to 0.65 (all *p* < 0.05); therefore, the mean of the three examiner ratings was used for analysis. Clinical, anatomical, prosthesis-related data and cosmetic examiner-rated scores are summarized in Table S1. Univariate correlations with cosmetic outcomes are provided in Table S2. Standard residual diagnostics did not indicate substantial heteroscedasticity, systematic model misspecification, or departures from residual normality of a magnitude suggesting that the linear regression models were inappropriate. Across the final integrated models, VIFs ranged from 1.04 to 2.54, indicating that there was no substantial multicollinearity. Final complete-case models included 99, 95, 92, and 85 patients for eyelid symmetry, orbital fullness, prosthesis recognizability, and general cosmetic appearance, respectively.

Comparisons of patients included and excluded from the final complete-case models showed no consistent differences in age, sex, etiology, orbital implant status, or history of additional periocular surgery. Excluded patients tended to have a longer duration of anophthalmia, reaching statistical significance for the eyelid-symmetry and general-appearance models (Table S3).

### 3.1. Eyelid Symmetry

In the blockwise model (Table 1), the strongest explanatory power for examiner-rated eyelid symmetry was attributed to prosthesis alignment and motility (R^2^ = 0.55), followed by socket and eyelid parameters (R^2^ = 0.49), clinical background (R^2^ = 0.19), and prosthesis structural features (R^2^ = 0.13). When the four block models were repeated in the same complete-case subset (*n* = 86), the corresponding adjusted R^2^ values were 0.14, 0.43, 0.55, and 0.17 for blocks A–D, respectively, preserving the same overall pattern.

In the integrated multiple regression, including only variables that remained significant within their respective blocks, two predictors retained independent associations: levator function (β = 0.19) and prosthesis motility (β = 0.68). The final model explained 61% of the variance (adjusted R^2^ = 0.59).

MRD1 showed no significant univariate association with the cosmetic rating of symmetry and was thus not included in the regression (Table S2). The distribution of MRD1 differences relative to the contralateral eye was narrowly clustered, with a median of 0 mm, an interquartile range (IQR) of 0–1 mm, and a range of −2 to +5 mm. Only 16 patients had relative ptosis. This restricted range may have limited the ability to detect associations at greater degrees of eyelid malposition.

Similarly, lower eyelid laxity compared to the contralateral eye was not significantly associated with eyelid symmetry in the univariate analysis. Only 20 patients (20%) had relatively lower eyelid laxity on the prosthesis side. Among them, the mean eyelid symmetry score was 3.3 ± 0.2 compared to 3.3 ± 0.1 in patients without increased laxity (*p* = 0.85).

Finally, prosthesis morphology (volume, thickness, posterior apex height) showed no association with the symmetry score in the regression (Table 1).

### 3.2. Orbital Fullness

In blockwise regression analysis (Table 2), socket and eyelid parameters showed the highest R^2^ in the original blockwise analysis for examiner-rated orbital fullness (R^2^ = 0.32), followed by prosthesis motility (R^2^ = 0.27), background characteristics (R^2^ = 0.17), and prosthesis structural features (R^2^ = 0.04). In the common complete-case subset (*n* = 86), adjusted R^2^ values were 0.16, 0.24, 0.24, and 0.00 for blocks A–D, respectively, indicating similar explanatory performance of the socket/eyelid and prosthesis motility blocks.

In the integrated model, only relative exophthalmometry (β = 0.27) and prosthesis motility (β = 0.34) remained significant predictors, explaining 34% of the variance in fullness scores (adjusted R^2^ = 0.32).

The relative exophthalmometry difference (anophthalmic minus contralateral eye) had a median of −2 mm (IQR −3 to −1 mm), indicating that the prosthetic side was typically enophthalmic. At least 2 mm of enophthalmos was present on the prosthetic side in 52% of patients (Table S1). Among these, the mean fullness score was 3.0 ± 0.2, compared to 3.7 ± 0.2 in patients with relative axial symmetry (*p* = 0.03).

Although both showed significant correlations in their respective blocks, neither orbital implant status nor socket support contour remained significant in the final model. Prosthesis volume, thickness, and height again failed to contribute meaningfully to perceived orbital fullness (Table 2).

### 3.3. Recognizability

In the regression modeling (Table 3), socket and eyelid parameters showed the highest R^2^ in the original blockwise analysis for examiner-rated prosthesis recognizability (R^2^ = 0.52), followed by prosthesis alignment and motility (R^2^ = 0.41), background characteristics (R^2^ = 0.27), and prosthesis structural features (R^2^ = 0.10). In the common complete-case subset (*n* = 77), adjusted R^2^ values were 0.26, 0.46, 0.46, and 0.07 for blocks A–D, respectively, indicating comparable explanatory performance of the socket/eyelid and alignment/motility blocks.

In the integrated model, only prosthesis motility (β = 0.55) and history of additional periocular surgeries (β = −0.22) remained significant predictors, explaining 46% of the variance (adjusted R^2^ = 0.43).

Patients with a history of additional corrective periocular surgeries had a significantly lower recognizability score, indicating greater prosthesis visibility than those without such a history (mean 2.2 ± 0.3 vs. 2.9 ± 0.1, *p* = 0.008). Furthermore, 28.6% (4/14) of patients in this group had visible external scars, compared with only 5% (4/84) among those who had not undergone further surgeries (*p* = 0.003).

Socket support and presence of an orbital implant, though significant in blockwise analysis, did not retain predictive value in the integrated multivariate model (Table 3). However, both were strongly associated with motility in univariate comparisons: patients with rounded socket support had higher mean motility scores (3.3 ± 0.1) compared to those with flat contour (2.4 ± 0.1, *p* < 0.0001); similarly, those with orbital implants had higher motility scores than those without (3.4 ± 0.1 vs. 2.6 ± 0.1, *p* < 0.0001). These findings suggest that their associations with prosthesis recognizability may be partly related to their associations with dynamic function.

Structural features of the prosthesis, including volume, projection, and thickness, were not significantly associated with recognizability scores.

### 3.4. General Cosmetic Appearance

In the blockwise regressions (Table 4), socket and eyelid parameters again showed the highest explanatory power for examiner-rated general cosmetic appearance (R^2^ = 0.65), followed by prosthesis alignment and motility (R^2^ = 0.46), background characteristics (R^2^ = 0.21), and prosthesis structural features (R^2^ = 0.16). In the common complete-case subset (*n* = 80), adjusted R^2^ values were 0.18, 0.62, 0.48, and 0.13 for blocks A–D, respectively, preserving the greater explanatory performance of the socket/eyelid block.

In the integrated model, only lagophthalmos (β = −0.38) and prosthesis motility (β = 0.35) retained significance. The final model explained 69% of the variance in general appearance scores (adjusted R^2^ = 0.66).

Other predictors, including additional periorbital surgeries, orbital implant status, levator function, socket contour, inferior fornix position, and prosthesis volume, failed to retain independent associations in the final model, despite block-level statistical significance (Table 4).

Levator function was highly correlated with lagophthalmos (r = −0.66, *p* < 0.0001); patients with good levator function had a mean lagophthalmos of 0.5 ± 0.2 mm compared to 6.7 ± 0.4 mm among those with poor function (*p* < 0.0001), suggesting collinearity and shared explanatory power in relation to perceived cosmetic outcomes.

## 4. Discussion

This study identified which clinical and prosthetic features are most strongly associated with cosmetic impressions in our cohort of ocular prosthesis wearers. Previous work has largely focused on patient-reported experience or complications, whereas few studies have systematically examined the clinical and prosthetic features associated with how others judge appearance [1,2,3,4,7,15,16,17,19,20,21,22].

Overall, the sensitivity analyses supported the same clinical pattern, with cosmetic appearance being more closely associated with eyelid/socket characteristics and prosthesis movement than with background factors or prosthesis dimensions. At the variable level, prosthesis motility and levator function were the primary independent predictors of eyelid symmetry; exophthalmometry (axial symmetry) and motility predicted orbital fullness; motility and additional periocular surgery predicted recognizability; and lagophthalmos and motility predicted overall appearance. Overall, the findings suggest that dynamic characteristics, especially those related to eyelid function and prosthesis movement, are more strongly associated with cosmetic judgments than static structural or background factors.

Our findings reinforce the long-recognized importance of prosthesis motility in cosmetic rehabilitation of the anophthalmic socket. Numerous studies have sought to enhance motility through variations in implant design, material, and surgical technique, including pegging [22,23,24,25]. Consistent with these efforts, patient-reported outcome studies likewise identify motility as a key determinant of cosmetic satisfaction [8,10,11,16,19]. In our analysis, motility was the only variable that independently predicted all four appearance domains.

In this study, clinical raters graded motility on a five-point horizontal amplitude scale, ranging from absent to near-normal movement. Although practical, this approach is inherently semi-subjective and may be influenced by the examiner’s overall impression. Nonetheless, motility remained an independent predictor across all appearance domains, even those without an obvious mechanistic link, such as orbital fullness or eyelid symmetry. This pattern suggests that perceived motility serves as a global visual cue of naturalness, shaping overall cosmetic judgments and influencing components that may be inseparable in the observer’s perception.

The semi-subjective nature of our measurement can be seen as both a limitation and a strength. In addition, the motility assessment was limited to horizontal excursion and did not capture vertical or more complex dynamic movements. Although it does not precisely quantify true prosthetic movement, cosmetic impressions in the real world depend on perceived motion rather than measured amplitude. Our findings may therefore better reflect the real-world association between perceived motility and appearance. Nevertheless, objective assessments—such as photographic analysis, infrared oculography, or gaze-tracking [26]—would help determine whether perceived and actual motility show similar associations with cosmetic evaluation.

Examiner-rated cosmetic appearance and patient-reported satisfaction are related but not interchangeable constructs, and the present analysis specifically addresses the former [7]. In clinical practice, attention to prosthesis motility and eyelid dynamics may help identify visually apparent contributors to cosmetic concerns, including in settings where formal quantitative motion analysis is not available.

Smerdon and Sutton [15] previously attempted to systematically relate examiner-rated cosmetic outcomes to clinical features, studying 52 enucleated patients. They identified upper-lid sulcus deformity as the sole factor associated with poorer appearance, whereas implant status and prosthesis dimensions showed no significant effects. Although limited by sample size and the absence of multivariable analysis, their observations stressed the relevance of orbital fullness [7]. Our study adds the finding that dynamic features—particularly prosthesis motility and upper-eyelid dynamics—show independent associations with cosmetic outcomes beyond static volume or fullness parameters.

The association between additional periocular surgery and greater prosthesis recognizability may partly reflect visible external cues, as scars were significantly more frequent in patients who had undergone additional procedures. However, additional surgery may also serve as a marker of greater pre-existing socket or periocular abnormality, which could not be assessed retrospectively. Etiology of anophthalmia may also influence the residual socket and periocular anatomy, particularly in patients with prior trauma; however, the cause of anophthalmia was not independently associated with the cosmetic outcomes in the present analyses.

Orbital implants are designed to restore volume (thereby diminishing sulcus deformity) and to transmit socket motility to the prosthesis [17,27]. Although implant status showed univariate associations with all cosmetic domains, these associations were lost in the multivariable models. This should not be interpreted as indicating that implants are unimportant to cosmetic rehabilitation; their contribution may be expressed through restoration of orbital volume and socket support, transmission of movement to the prosthesis, and other downstream anatomical and functional effects represented by variables included in the multivariable models [21,27,28,29].

A longstanding view holds that compensating for ptosis or orbital volume loss with a larger or heavier prosthesis may induce secondary changes such as lower-lid laxity or superior sulcus deformity [15,17]. Smerdon and Sutton [15] found no correlation between prosthesis weight and these features, and prosthesis dimensions similarly did not retain independent associations with cosmetic outcomes in our multivariable models. Overall, our findings indicate that appearance is more strongly associated with the dynamic interaction between orbital anatomy, soft-tissue adaptation, and eyelid and prosthesis movement than with prosthesis size or static volume restoration.

Several limitations should be acknowledged. First, this single-center study included mostly uncomplicated prosthesis wearers attending routine follow-up at a tertiary ocular prosthetics clinic, which may limit generalizability to other settings and to patients with more complex socket sequelae. The cross-sectional design does not allow temporal relationships or causal effects to be established. The relatively narrow range of some anatomical abnormalities may also have reduced our ability to detect associations that could be more apparent in patients with severe socket or eyelid deformity. Detailed surgical information—including type of eye-removal surgery, implant material, and specific prior reconstructive procedures—was not systematically recorded and therefore could not be incorporated into the present analyses. The sample size supported meaningful multivariable analysis but may still have been underpowered to detect weaker associations. Selection bias related to missing data cannot be entirely excluded, particularly as excluded patients had a longer duration of anophthalmia in some models. Cosmetic assessments were examiner-based and, although structured, not fully standardized; however, agreement among three independent observers likely mitigated individual bias. Nonetheless, because the same examiners rated prosthesis motility and cosmetic appearance, common-rater bias cannot be excluded, particularly for associations between perceived motility and cosmetic ratings. Next, the multistage modeling strategy was based on predefined clinical domains and designed to limit the number of variables entering the integrated models; however, as with data-driven variable selection, the resulting models should be considered exploratory and require validation in independent cohorts. Prosthesis volume was measured by water displacement, which may be less precise than three-dimensional measurement techniques. Finally, all participants used custom-made acrylic (PMMA) prostheses, so the findings may not generalize to other prosthesis materials or fabrication approaches. In particular, cryolite glass prostheses differ in surface and optical properties [9,30]. Cosmetic impressions may therefore vary across materials [9], warranting dedicated comparative studies.

## 5. Conclusions

In conclusion, dynamic anatomical features—particularly prosthesis motility and upper-eyelid function—showed the strongest associations with cosmetic appearance after eye loss. Prosthesis motility was the most consistent predictor, showing independent associations with all appearance domains, while upper-eyelid function was associated with symmetry and overall impression. Static features such as implant status or prosthesis dimensions showed comparatively limited independent associations once dynamics were considered. In clinical follow-up, attention to motility and eyelid dynamics may therefore be most useful when addressing appearance-related concerns. From an outcome-assessment perspective, these findings also suggest that cosmetic evaluation after eye loss should incorporate dynamic features rather than rely predominantly on static anatomical measures. Future work using objective motion analysis could clarify whether true motility deficits and perceived movement affect cosmetic judgment similarly.

## Figures and Tables

**Table 1:** Blockwise multiple regression analysis for eyelid symmetry. Final integrated model: R^2^ = 0.61; adjusted R^2^ = 0.59; *n* = 99.

Block	Predictor	Category	Block Model β	Block Model *p*	Final Integrated Model β	Final Integrated Model *p*
A. Clinical background (R^2^ = 0.19; adjusted R^2^ = 0.16; *n* = 104)						
	Age (Years)		−0.07	0.49		
	Time with anophthalmia (Years)		−0.12	0.27		
	Orbital implant	Yes	**0.34**	**<0.001**	0.06	0.49
		*No*				
B. Socket and eyelid (R^2^ = 0.49; adjusted R^2^ = 0.44; *n* = 88)						
	Levator function (mm)		**0.29**	**0.011**	**0.19**	**0.020**
	Lagophthalmos (mm)		−0.11	0.50		
	Socket support	Rounded	**0.24**	**0.007**	0.07	0.42
		*Flat*				
	Inferior fornix depth (mm)		0.05	0.55		
	Superior fornix depth (mm)		0.01	0.91		
	Inferior fornix position (relative to inferior rim)	Anterior	−0.04	0.67		
		*Posterior*				
	Socket/conjunctival contracture score		0.20	0.19		
C. Prosthesis alignment and motility (R^2^ = 0.55; adjusted R^2^ = 0.54; *n* = 106)						
	Prosthesis motility score		**0.71**	**<0.0001**	**0.68**	**<0.0001**
	Prosthesis primary gaze alignment	Yes	0.10	0.15		
		*No*				
D. Prosthesis structural dimensions (R^2^ = 0.13; adjusted R^2^ = 0.11; *n* = 106)						
	Central thickness (T, mm)		−0.12	0.47		
	Posterior apex height (D, mm)		0.28	0.16		
	Posterior projection index (D/T)		0.01	0.97		

β values are standardized regression coefficients. Block β and *p* values are from the corresponding domain-specific model; final β and *p* values are from the integrated model. **Boldface** indicates *p* < 0.05. *Italicized categories indicate the reference category.*

**Table 2:** Blockwise multiple regression analysis for orbital fullness. Final integrated model: R^2^ = 0.34; adjusted R^2^ = 0.32; *n* = 95.

Block	Predictor	Category	Block Model β	Block Model *p*	Final Integrated Model β	Final Integrated Model *p*
A. Clinical background (R^2^ = 0.17; adjusted R^2^ = 0.14; *n* = 97)						
	Age (Years)		−0.15	0.18		
	Time with anophthalmia (Years)		−0.05	0.66		
	Orbital implant	Yes	**0.32**	**0.003**	0.07	0.58
		*No*				
B. Socket and eyelid (R^2^ = 0.32; adjusted R^2^ = 0.26; *n* = 88)						
	Levator function (mm)		0.13	0.31		
	Lagophthalmos (mm)		−0.22	0.27		
	Exophthalmometry (vs. fellow eye, mm)		**0.25**	**0.025**	**0.27**	**0.039**
	Socket support	Rounded	**0.27**	**0.010**	0.14	0.26
		*Flat*				
	Inferior fornix depth (mm)		0.03	0.80		
	Superior fornix depth (mm)		0.11	0.40		
	Inferior fornix position (relative to inferior rim)	Anterior	−0.05	0.63		
		*Posterior*				
	Socket/conjunctival contracture score		0.06	0.75		
C. Prosthesis alignment and motility (R^2^ = 0.27; adjusted R^2^ = 0.26; *n* = 99)						
	Prosthesis motility score		**0.52**	**<0.0001**	**0.34**	**0.002**
D. Prosthesis structural dimensions (R^2^ = 0.04; adjusted R^2^ = 0.01; *n* = 99)						
	Central thickness (T, mm)		−0.03	0.88		
	Posterior apex height (D, mm)		0.14	0.51		
	Posterior projection index (D/T)		0.07	0.82		

β values are standardized regression coefficients. Block β and *p* values are from the corresponding domain-specific model; final β and *p* values are from the integrated model. **Boldface** indicates *p* < 0.05. *Italicized categories indicate the reference category.*

**Table 3:** Blockwise multiple regression analysis for prosthesis recognizability. Final integrated model: R^2^ = 0.46; adjusted R^2^ = 0.43; *n* = 92.

Block	Predictor	Category	Block Model β	Block Model *p*	Final Integrated Model β	Final Integrated Model *p*
A. Clinical background (R^2^ = 0.27; adjusted R^2^ = 0.22; *n* = 93)						
	Age (Years)		−0.06	0.51		
	Time with anophthalmia (Years)		**−0.27**	**0.018**	−0.12	0.19
	Cause of anophthalmia	Trauma	−0.05	0.72		
		Tumor	0.05	0.70		
		*Disease*				
	Additional periorbital surgeries	Yes	**−0.30**	**0.002**	**−0.22**	**0.008**
		*No*				
	Orbital implant	Yes	**0.30**	**0.003**	0.06	0.60
		*No*				
B. Socket and eyelid (R^2^ = 0.52; adjusted R^2^ = 0.48; *n* = 87)						
	Levator function (mm)		0.19	0.08		
	Lagophthalmos (mm)		−0.12	0.48		
	Socket support	Rounded	**0.25**	**0.004**	0.08	0.51
		*Flat*				
	Inferior fornix depth (mm)		0.06	0.48		
	Superior fornix depth (mm)		0.02	0.86		
	Inferior fornix position (relative to inferior rim)	Anterior	−0.12	0.20		
		*Posterior*				
	Socket/conjunctival contracture score		0.24	0.11		
C. Prosthesis alignment and motility (R^2^ = 0.41; adjusted R^2^ = 0.40; *n* = 105)						
	Prosthesis motility score		**0.62**	**<0.0001**	**0.55**	**<0.0001**
	Prosthesis primary gaze alignment	Yes	0.08	0.31		
		*No*				
D. Prosthesis structural dimensions (R^2^ = 0.10; adjusted R^2^ = 0.08; *n* = 105)						
	Central thickness (T, mm)		−0.14	0.41		
	Posterior apex height (D, mm)		0.27	0.19		
	Posterior projection index (D/T)		0.04	0.90		

β values are standardized regression coefficients. Block β and *p* values are from the corresponding domain-specific model; final β and *p* values are from the integrated model. **Boldface** indicates *p* < 0.05. *Italicized categories indicate the reference category.*

**Table 4:** Blockwise multiple regression analysis for general cosmetic appearance. Final integrated model: R^2^ = 0.69; adjusted R^2^ = 0.66; *n* = 85.

Block	Predictor	Category	Block Model β	Block Model *p*	Final Integrated Model β	Final Integrated Model *p*
A. Clinical background (R^2^ = 0.21; adjusted R^2^ = 0.17; *n* = 97)						
	Age (Years)		−0.01	0.90		
	Time with anophthalmia (Years)		−0.21	0.052		
	Additional periorbital surgeries	Yes	**−0.29**	**0.002**	−0.01	0.92
		*No*				
	Orbital implant	Yes	**0.22**	**0.029**	0.07	0.45
		*No*				
B. Socket and eyelid (R^2^ = 0.65; adjusted R^2^ = 0.62; *n* = 88)						
	Levator function (mm)		**0.23**	**0.015**	0.13	0.17
	Lagophthalmos (mm)		**−0.31**	**0.031**	**−0.38**	**<0.001**
	Exophthalmometry (vs. fellow eye, mm)		0.08	0.28		
	Socket support	Rounded	**0.16**	**0.032**	0.02	0.84
		*Flat*				
	Inferior fornix depth (mm)		0.02	0.79		
	Superior fornix depth (mm)		0.01	0.88		
	Inferior fornix position (relative to inferior rim)	Anterior	**−0.17**	**0.039**	−0.12	0.13
		*Posterior*				
	Socket/conjunctival contracture score		0.13	0.33		
C. Prosthesis alignment and motility (R^2^ = 0.46; adjusted R^2^ = 0.45; *n* = 106)						
	Prosthesis motility score		**0.65**	**<0.0001**	**0.35**	**<0.001**
	Prosthesis primary gaze alignment	Yes	0.10	0.20		
		*No*				
D. Prosthesis structural dimensions (R^2^ = 0.16; adjusted R^2^ = 0.13; *n* = 105)						
	Volume (ml)		**0.27**	**0.032**	0.08	0.23
	Central thickness (T, mm)		−0.13	0.48		
	Posterior apex height (D, mm)		0.19	0.36		
	Posterior projection index (D/T)		0.03	0.90		

β values are standardized regression coefficients. Block β and *p* values are from the corresponding domain-specific model; final β and *p* values are from the integrated model. **Boldface** indicates *p* < 0.05. *Italicized categories indicate the reference category.*

## Data Availability

The anonymized data supporting the findings of this study are available from the corresponding author upon reasonable request.

## Supplementary Materials

The following supporting information can be downloaded at https://www.mdpi.com/article/10.3390/jcm15208054/s1. Table S1: Descriptive statistics of demographic, clinical, anatomical, and prosthesis-related baselines as well as examiner-rated cosmetic scores in 107 anophthalmic patients with artificial eyes. Table S2: Univariate analysis of the association of clinical, eyelid, socket, and prosthesis-related parameters with examiner-rated cosmetic scores in 107 anophthalmic patients with an artificial eye. Table S3: Baseline characteristics of patients included in and excluded from the final complete-case models.

## References

[1] Ruiters S., Mombaerts I. (2021). The prevalence of anophthalmic socket syndrome and its relation to patient quality of life. Eye.

[2] Johnson T.E. (2020). Management of the Contracted Socket. Anophthalmia.

[3] Levitt A.E., Lee B.W. (2020). Anophthalmic Socket Syndrome. Anophthalmia.

[4] Rokohl A.C., Trester M., Pine K.R., Heindl L.M. (2020). Prevention of Socket Complications in Anophthalmic Patients. Curr. Eye Res..

[5] Rokohl A.C., Kopecky A., Trester M., Matos P.A.W., Pine K.R., Heindl L.M. (2022). Post-enucleation socket syndrome—A novel pathophysiological definition. Graefe’s Arch. Clin. Exp. Ophthalmol..

[6] Wadhwa A.B., Patel P.K., Zhao L., Cohen M., Brown R.J.B., Arias-Amezquita E.M. (2025). Visual Assessment of Facial Differences in Patients Reconstructed with Ocular Prostheses: A Web-Based Survey. J. Craniofacial Surg..

[7] Vardizer Y., Sobeh T., Prat D.L., Ben Simon G.J., Tomkins-Netzer O. (2021). Assessing the results of anophthalmic prostheses. Indian J. Ophthalmol..

[8] Shapira Y., Worrell E., Litwin A.S., Malhotra R. (2021). The UK National Artificial Eye Questionnaire study: Predictors of artificial eye wearers’ experience part 1—Comfort and satisfaction. Eye.

[9] Rokohl A.C., Koch K.R., Adler W., Trester M., Trester W., Pine N.S., Pine K.R., Heindl L.M. (2018). Concerns of anophthalmic patients—A comparison between cryolite glass and polymethyl methacrylate prosthetic eye wearers. Graefe’s Arch. Clin. Exp. Ophthalmol..

[10] Pine N.S., de Terte I., Pine K.R. (2017). An investigation into discharge, visual perception, and appearance concerns of prosthetic eye wearers. Orbit.

[11] Song J.S., Oh J., Baek S.H. (2006). A survey of satisfaction in anophthalmic patients wearing ocular prosthesis. Graefe’s Arch. Clin. Exp. Ophthalmol..

[12] James H., Jenkinson E., Harrad R., Ezra D.G., Newman S. (2011). Appearance concerns in ophthalmic patients. Eye.

[13] Pine K., Sloan B., Stewart J., Jacobs R.J. (2011). Concerns of anophthalmic patients wearing artificial eyes. Clin. Exp. Ophthalmol..

[14] Hatamleh M.M., Alnazzawi A.A., Abbariki M., Alqudah N., Cook A.E. (2017). Survey of Ocular Prosthetics Rehabilitation in the United Kingdom, Part 2. J. Craniofacial Surg..

[15] Smerdon D.L., Sutton G.A. (1988). Analysis of the factors involved in cosmetic failure following excision of the eye. Br. J. Ophthalmol..

[16] Dave T.V., Nayak A., Palo M., Goud Y., Tripuraneni D., Gupta S. (2021). Custom ocular prosthesis-related concerns: Patient feedback survey-based report vis-à-vis objective clinical grading scales. Orbit.

[17] Jordan D.R., Klapper S.R. (2021). Evaluation and Management of the Anophthalmic Socket and Socket Reconstruction. Smith and Nesi’s Ophthalmic Plastic and Reconstructive Surgery.

[18] Tabachnick B.G., Fidell L.S. (2019). Using Multivariate Statistics.

[19] van Renterghem V., Ruiters S., Mombaerts I. (2023). Motility of the Ocular Prosthesis in Anophthalmic Patients: Objective and Patient-perceived Findings. Orbit.

[20] Dave T.V. (2021). Commentary: Assessing the results of anophthalmic prostheses. Indian J. Ophthalmol..

[21] Custer P.L., Maamari R.N., Huecker J.B., Gordon M.O. (2021). Anophthalmic Ptosis and the Effects of Enucleation on Upper Eyelid Function. Ophthalmic Plast. Reconstr. Surg..

[22] Bigheti C.P., Peitl O., Ferreira G.d.A., Schellini S.A. (2022). Amplitude of movements with conical or spherical implants in anophthalmic socket. Orbit.

[23] Yom K.H., Ko A.C. (2020). History of Ocular Implants. Anophthalmia.

[24] Wladis E.J., Aakalu V.K., Sobel R.K., Yen M.T., Bilyk J.R., Mawn L.A. (2018). Orbital Implants in Enucleation Surgery. Ophthalmology.

[25] Custer P.L., Kennedy R.H., Woog J.J., Kaltreider S.A., Meyer D.R. (2003). Orbital implants in enucleation surgery. Ophthalmology.

[26] Mourits D.L., Hartong D.T., van Beek J.H.M., Witte B.I., Tan H.S., Moll A.C. (2017). A Novel Method to Measure Artificial Eye Motility. Ophthalmic Plast. Reconstr. Surg..

[27] Custer P.L., Maamari R.N., Huecker J.B., Gordon M.O. (2021). Volumetric Versus Empirical Determination of Enucleation Implant Size. Ophthalmic Plast. Reconstr. Surg..

[28] Blessing N.W. (2020). Socket Inflammation and Infection. Anophthalmia.

[29] Kaltreider S.A., Jacobs J.L., Hughes M.O. (1999). Predicting the Ideal Implant Size Before Enucleation. Ophthalmic Plast. Reconstr. Surg..

[30] Rokohl A.C., Pine K.R., Pine N.S., Gordon E., Yeoman J., Remmers J.S., Hartong D.T., Heindl L.M. (2025). Prosthetic eye care—The current state of the art. Prog. Retin. Eye Res..

