# Supplementary material for: Cosmetic Appearance After Eye Loss: The Role of Prosthesis Motility and Eyelid Function
Source: J Clin Med. 2026 Oct 16;15(20):8054. doi: 10.3390/jcm15208054 (PMC13706109; doi:10.3390/jcm15208054)
Supplement: Supplementary file 1 [file jcm-15-08054-s001.zip › jcm-4585846-supplementary.pdf]

Table S1: Descriptive statistics of demographic, clinical, anatomical, and prosthesis-related baselines as well as examiner-rated cosmetic scores in 107 anophthalmic patients with artificial eyes

Parameter	Category	N (%) / Mean ± SD	Range (IQR)	Missing n
Age (years)		53.1 ± 18.6	18–89 (37–70)	1
Time with anophthalmia (years)		25.5 ± 21.0	1.0–76.0 (6.8–39.2)	3
Side	Right	47 (45.2%)		
	Left	57 (54.8%)		3
Gender	Male	52 (49.1%)		
	Female	54 (50.9%)		1
Birthplace	Native	54 (50.9%)		
	Foreign-born	52 (49.1%)		1
Religion	Jewish	80 (76.2%)		
	Muslim	15 (14.3%)		
	Christian	5 (4.8%)		
	Other	5 (4.8%)		2
General health	No comorbidities	64 (64.0%)		
	Comorbidities	36 (36.0%)		7
Etiology	Trauma	50 (49.5%)		
	Ocular disease	41 (40.6%)		
	Ocular malignancy	10 (9.9%)		6
Orbital implant present	Yes	46 (43.4%)		
	No	60 (56.6%)		1
Additional periocular surgeries	Yes	14 (14.3%)		
	No	84 (85.7%)		9
MRD1 (relative, mm)		0.5 ± 1.3	-2.0–5.0 (0.0–1.0)	8
Levator function (mm)		9.8 ± 4.3	0.0–20.0 (7.0–12.0)	4
Levator function grade	Good	71 (68.9%)		
	Fair	17 (16.5%)		
	Poor	15 (14.6%)		4
Lagophthalmos (mm)		1.5 ± 2.6	0.0–11.7 (0.0–2.2)	1
Lower eyelid laxity (relative)	Yes	20 (20.0%)		7

	No	80 (80.0%)		
Exophthalmometry (relative, mm)		-1.9 ± 3.1	-12–11 (-3–-1)	5
Prosthesis axial position (relative)	Enophthalmos ≥2 mm	53 (52.0%)		
	Exophthalmos ≥2 mm	8 (7.8%)		5
Inferior fornix depth (mm)		7.7 ± 2.5	2.0–21.0 (6.0–9.0)	5
Superior fornix depth (mm)		16.4 ± 4.5	1.0–27.0 (14.0–20.0)	5
Inferior fornix position (relative to inferior rim)	Anterior	8 (8.1%)		
	Posterior	91 (91.9%)		8
Socket support (rounded contour)	Yes	58 (56.9%)		
	No	44 (43.1%)		5
Socket/conjunctival contracture		2.7 ± 0.6	1.0–3.0 (2.8–3.0)	8
Prosthesis motility		2.9 ± 0.9	1.0–5.0 (2.0–3.5)	1
Prosthesis primary gaze alignment	Aligned	96 (90.6%)		
	Misaligned	10 (9.4%)		1
Prosthesis volume (mL)		2.6 ± 1.2	0.5–6.0 (2.0–3.5)	2
Prosthesis central thickness (T, mm)		8.0 ± 2.2	3.0–12.5 (7.0–9.5)	1
Posterior prosthesis height (D, mm)		4.9 ± 2.8	0.0–19.0 (3.0–6.0)	1
Posterior projection index (D/T)		0.72 ± 0.54	0.00–3.00 (0.34–0.94)	1
Eyelid symmetry score		3.2 ± 0.9	1.0–5.0 (2.6–4.0)	1
Orbital fullness score		3.3 ± 1.2	1.0–5.0 (2.5–4.5)	8
Prosthesis recognizability score		2.8 ± 1.0	1.0–4.0 (2.0–3.5)	2
General cosmetic score		7.0 ± 2.1	1.5–10.0 (6.0–8.5)	1

Table S2: Univariate analysis of the association of clinical, eyelid, socket, and prosthesis-related parameters with examiner-rated cosmetic scores, in 107 anophthalmic patients with an artificial eye

		**Eyelid symmetry**		**Orbital fullness**		**Prosthesis recognizability**		**General cosmetic appearance**	
	**Variable**	**r[1]**	**p-value[2]**	**r[1]**	**p-value[2]**	**r[1]**	**p-value[2]**	**r[1]**	**p-value[2]**
Demographics / Clinical Background	Age (Years)	**-0.28**	**0.004**	**-0.26**	**0.010**	**-0.18**	**0.067**	**-0.20**	**0.040**
	Gender		0.56		0.30		0.43		0.54
	Birthplace (immigration)		0.15		0.21		0.64		0.41
	Religion		0.35		0.52		0.77		0.27
	General health		0.45		0.42		0.64		0.45
	Eye		0.97		0.93		0.58		0.99
	Cause of anophthalmia		0.57		0.73		**0.099**		0.71
	Time with anophthalmia (Years)	**-0.32**	**0.001**	**-0.24**	**0.018**	**-0.35**	**<0.001**	**-0.29**	**0.002**
	Orbital implant present		**<0.0001**		**<0.001**		**<0.001**		**<0.001**
	Additional periorbital surgeries		0.21		0.53		**0.038**		**0.056**
Socket and Eyelid	MRD1 (vs. fellow eye, mm)	0.07	0.51	-0.01	0.92	0.10	0.32	0.09	0.37

	Levator function (mm)	**0.46**	**<0.0001**	**0.46**	**<0.0001**	**0.48**	**<0.0001**	**0.55**	**<0.0001**
	Lagophthalmos (mm)	**-0.41**	**<0.0001**	**-0.32**	**0.002**	**-0.47**	**<0.0001**	**-0.55**	**<0.0001**
	Lower eyelid laxity		0.86		0.79		0.37		0.77
	Exophthalmometry (vs. fellow eye, mm)	0.01	0.94	**0.35**	**<0.001**	0.10	0.30	**0.22**	**0.024**
	Socket support		**<0.0001**		**<0.001**		**<0.0001**		**<0.0001**
	Inferior fornix depth (mm)	**0.30**	**0.002**	**0.19**	**0.057**	**0.28**	**0.004**	**0.28**	**0.004**
	Superior fornix depth (mm)	**0.26**	**0.008**	**0.17**	**0.091**	**0.31**	**0.002**	**0.33**	**<0.001**
	Inferior fornix position		**0.004**		**0.095**		**<0.001**		**<0.001**
	Socket contracture	**0.50**	**<0.0001**	**0.36**	**<0.001**	**0.53**	**<0.0001**	**0.51**	**<0.0001**
Prosthesis alignment and motility	Prosthesis motility	**0.72**	**<0.0001**	**0.51**	**<0.0001**	**0.66**	**<0.0001**	**0.66**	**<0.0001**
	Prosthesis primary gaze alignment		**0.022**		0.26		**0.025**		**0.018**
Prosthesis Morphology	Volume (ml)	0.12	0.22	0.11	0.27	0.10	0.32	**0.16**	**0.10**
	Central thickness (T, mm)	**-0.34**	**<0.001**	**-0.19**	**0.057**	**-0.31**	**0.001**	**-0.24**	**0.014**
	Posterior apex height (D, mm)	**0.47**	**<0.0001**	**0.27**	**0.008**	**0.39**	**<0.0001**	**0.43**	**<0.0001**
	Posterior projection index (D/T)	**0.45**	**<0.0001**	**0.24**	**0.019**	**0.37**	**<0.0001**	**0.37**	**<0.001**

[1] Spearman's r (applicable to continuous or ordinal parameters) [2]Spearman's rank correlation, Mann–Whitney U test, or the Kruskal–Wallis test, as applicable. Values in **boldface** mark parameters entered into the multiple regression models (p≤ 0.10)

Table S3. Baseline characteristics of patients included in and excluded from the final complete-case models

Baseline characteristic	A. Eyelid symmetry			B. Orbital fullness		
	Included (n = 99)	Excluded (n = 8)	p-value	Included (n = 95)	Excluded (n = 12)	p-value
Age, years	54.0 (36.0–70.5)<br>n = 99	55.0 (53.5–69.0)<br>n = 7	0.514	54.0 (36.5–69.0)<br>n = 95	66.0 (49.5–73.0)<br>n = 11	0.301
Duration of anophthalmia, years	17.0 (6.0–39.0)<br>n = 97	35.0 (29.5–46.0)<br>n = 7	**0.047**	17.0 (6.0–39.0)<br>n = 93	27.0 (18.0–43.5)<br>n = 11	0.179
**Sex**			1.000			0.759
Male	49/99 (49.5%)	3/7 (42.9%)		46/95 (48.4%)	6/11 (54.5%)	
Female	50/99 (50.5%)	4/7 (57.1%)		49/95 (51.6%)	5/11 (45.5%)	
**Etiology**			0.234			0.380
Trauma	47/94 (50.0%)	3/7 (42.9%)		45/91 (49.5%)	5/10 (50.0%)	
Ocular disease	39/94 (41.5%)	2/7 (28.6%)		38/91 (41.8%)	3/10 (30.0%)	
Ocular malignancy	8/94 (8.5%)	2/7 (28.6%)		8/91 (8.8%)	2/10 (20.0%)	
**Orbital implant**			0.136			0.753
Present	45/99 (45.5%)	1/7 (14.3%)		42/95 (44.2%)	4/11 (36.4%)	
Absent	54/99 (54.5%)	6/7 (85.7%)		53/95 (55.8%)	7/11 (63.6%)	
**Additional periocular surgery**			0.589			0.349
Yes	14/91 (15.4%)	0/7 (0.0%)		14/88 (15.9%)	0/10 (0.0%)	
No	77/91 (84.6%)	7/7 (100.0%)		74/88 (84.1%)	10/10 (100.0%)	

Table S3. Baseline characteristics of patients included in and excluded from the final complete-case models (continued)

Baseline characteristic	C. Prosthesis recognizability			D. General cosmetic appearance		
	Included (n = 92)	Excluded (n = 15)	p-value	Included (n = 85)	Excluded (n = 22)	p-value
Age, years	54.5 (36.0–70.0)<br>n = 92	54.0 (40.0–71.5)<br>n = 14	0.504	53.0 (35.0–66.0)<br>n = 85	65.0 (46.0–74.0)<br>n = 21	0.085
Duration of anophthalmia, years	18.5 (6.0–39.0)<br>n = 92	28.0 (16.0–67.0)<br>n = 12	0.091	16.0 (6.0–37.3)<br>n = 84	33.5 (19.8–52.8)<br>n = 20	**0.008**
**Sex**			1.000			0.090
Male	45/92 (48.9%)	7/14 (50.0%)		38/85 (44.7%)	14/21 (66.7%)	
Female	47/92 (51.1%)	7/14 (50.0%)		47/85 (55.3%)	7/21 (33.3%)	
**Etiology**			1.000			0.744
Trauma	44/89 (49.4%)	6/12 (50.0%)		40/83 (48.2%)	10/18 (55.6%)	
Ocular disease	36/89 (40.4%)	5/12 (41.7%)		35/83 (42.2%)	6/18 (33.3%)	
Ocular malignancy	9/89 (10.1%)	1/12 (8.3%)		8/83 (9.6%)	2/18 (11.1%)	
**Orbital implant**			0.089			0.051
Present	43/92 (46.7%)	3/14 (21.4%)		41/85 (48.2%)	5/21 (23.8%)	
Absent	49/92 (53.3%)	11/14 (78.6%)		44/85 (51.8%)	16/21 (76.2%)	
**Additional periocular surgery**			1.000			0.204
Yes	13/92 (14.1%)	1/6 (16.7%)		14/85 (16.5%)	0/13 (0.0%)	
No	79/92 (85.9%)	5/6 (83.3%)		71/85 (83.5%)	13/13 (100.0%)	

Values are presented as median (interquartile range) for continuous variables and count/available denominator (%) for categorical variables. Missing baseline values were omitted separately for each comparison and were not classified as "No." Included and excluded groups together comprise all 107 patients for each model. Continuous variables

were compared using the Mann–Whitney U test, binary categorical variables using Fisher's exact test, and etiology using the Fisher–Freeman–Halton exact test. All tests were two-sided, and $p < 0.05$ was considered statistically significant. Complete-case membership required observed values for the outcome and all predictors entered into the corresponding final integrated model. Abbreviation: n, number of patients.
